# Mitochondrial Phylogeography of Wild Boars, *Sus scrofa*, from Asia Minor: Endemic Lineages, Natural Immigration, Historical Anthropogenic Translocations, and Possible Introgression of Domestic Pigs

**DOI:** 10.3390/ani15131828

**Published:** 2025-06-20

**Authors:** Yasin Demirbaş, Hakan Soysal, Ayςa Özkan Koca, Milomir Stefanović, Franz Suchentrunk

**Affiliations:** 1 Department of Biology, Faculty of Engineering and Natural Sciences, Kırıkkale University, 71450 Yahşihan, Kırıkkale, Türkiye or yasdemirbas@kku.edu.tr (Y.D.); hsoysal50@msn.com (H.S.); 2 Department of Gastronomy and Culinary Arts, Faculty of Fine Arts, Maltepe University, 34857 Maltepe, İstanbul, Türkiye; aycakoca@maltepe.edu.tr; 3 Department of Biology and Ecology, Faculty of Sciences, University of Novi Sad, 21000 Novi Sad, Serbia; 4 Research Institute of Wildlife Ecology, University of Veterinary Medicine Vienna, 1160 Vienna, Austria

**Keywords:** wild boar, *Sus scrofa*, phylogeography, mtDNA, Middle East, domestic pig, introgression, historical/prehistorical translocations, introduction, gene flow

## Abstract

Türkiye represents an important biogeographic region connecting Southeast Europe with Southwest Asia, where pig domestication began in the western Palearctic. We studied the phylogenetic relationships and spatial distribution of new and published mitochondrial D-loop sequences of wild boars from Türkiye, other parts of the Middle East, and from around the world to understand migration patterns within Asia Minor and other parts of the Middle East as well as across the Bosphorus/Sea of Marmara/Dardanelles, a current migration barrier to Southwest Europe. Our phylogenetic (ML, BI) and spatial (Geneland) analyses revealed haplotypes both endemic to Anatolia and with a wider distribution in the Middle East as well as European (E1) lineages. The latter suggested possible rare immigration into Anatolia at present times and prehistorical/historical anthropogenic translocations of wild boars or pigs, such as during the pre-Hellenic, Roman, and Byzantine periods or during the European crusades, and subsequent introgression into Anatolian wild boars. Import of pigs with E1 haplotypes and introgression into wild boars during the medieval Empire of Trebizond particularly by Italian merchants or settlers, is also suggested. Anatolian lineages that may have formed the basis of the archaic domestication process of pigs in the western Palearctic are discussed.

## Simple Summary

Wild boars from the Middle East, particularly from northern Mesopotamia, formed the original basis of pig domestication at the end of the late Pleistocene. Later, in archaic times, during the classical Greek and Roman periods and the time of the Christian crusaders, wild boars or pigs of European origin were imported and may have interbred with local wild boars, with possible effects on phenotypes and genetic variants in wild boars in Anatolia. We studied the spatial distribution of maternally inherited (mitochondrial) DNA lineages of wild boars from Türkiye to determine whether only natural lineages are present or also lineages indicating historical introductions of pigs or wild boars specifically from Europe. We found native lineages and ones with wider distribution in the Middle East, but also (modern) pig lineages, suggesting prehistoric and/or historical introductions by humans. Our results also showed that the Bosphorus/Sea of Marmara/Dardanelles waterway between northwestern Anatolia and the southeastern Balkans currently represents an effective barrier to natural gene flow in wild boars, at least for females. Apparently, no relevant (maternal) gene flow occurred in the past, when a natural land bridge connected Anatolia with Southeast Europe. The Anatolian lineages are evolutionarily closely linked to other lineages from the Middle East, without a significant evolutionary gap.

## 1. Introduction

Asia Minor, i.e., the Anatolian Peninsula of Türkiye in Southwest Asia, together with the adjacent regions in the Middle East, the Caucasus, and the Southern Balkans in Europe, is considered as one of the larger biogeographic crossroads in the western Palearctic with rich biodiversity where Temperate Western Boreal, Eastern Mediterranean, Boreo-Siberian, African/Arabian, and Sindo-Turanian faunal elements meet [1,2]. It extends from south of the Caucasus to the west and to the northern part of the “Fertile Crescent”, i.e., northern Mesopotamia in southeastern Anatolia, and to the northern Levant, which connects to the Arabian Peninsula and northeastern Africa. In the west, it had a land bridge connection with the southeasternmost part of the Balkan Peninsula in Europe until the Middle Holocene before its final interruption ca. 8000–8500 YBP (years before present), due to the steadily raising postglacial sea level [3,4,5]. Its geographical location and its variable climate and landscape [6] have resulted in high inter- and intraspecific biological diversity [7,8], specifically also for terrestrial mammal species, which often reach their distribution limits either within the Anatolian Peninsula or in the west at the Bosphorus, the Sea of Marmara, and the Strait of Çanakkale (i.e., the Dardanelles—the ancient “Hellespont”), the region of the late Pleistocene/early Holocene land bridge, which today is obviously an effective barrier to migration for many terrestrial mammal species [9,10,11,12,13]. Brown hares, *Lepus europaeus*, for example, which have been used as a model for terrestrial mammal species to understand the potential effects of this region as a barrier to migration [14,15,16,17,18,19] and associated intraspecific evolutionary effects [20,21,22,23], have managed to expand their original Anatolian range westward into the southeastern Balkans during the late Pleistocene. In contrast, for badgers (*Meles* spp.), for example, such transcontinental migration was impossible and has obviously resulted in an effective separation of two different species on both sides of that biogeographic demarcation: i.e., *Meles meles* on the European side and *M. canescens* on the Asiatic side on the Anatolian Peninsula, as suggested by mitochondrial phylogeographic data [24].

Wild boars, *Sus scrofa*, are, on the one hand, capable dispersers over short distances and can also cross bodies of water by swimming. On the other hand, particularly family groups may maintain relatively restricted home ranges for extended periods of time, provided their habitat offers sufficient food, shelter, and safety from potential predators [25,26,27,28]. Although wild boars are considered ecologically quite adaptable, as is already suggested by their wide geographical distribution [28], as elsewhere, long-term changes in climate and habitat quality have probably strongly influenced their population histories and genetic composition in Türkiye [26,29]. Hence, the genetic composition of wild boars from the Anatolian Peninsula may have resulted from both the presence of regionally longer existing (endemic?) gene pools and gene flow from the southeast, the east, the northeast, as well as from SE Europe across the western late Pleistocene/early Holocene land bridge.

Furthermore, the (northern) Fertile Crescent was the region where the first wild boar management measures may have originated in pre-Neolithic times, probably as early as ca. 12,000 YBP [30]. Free-range pig farming over thousands of generations and/or escapes of pigs from prehistoric farms or husbandry units ([30,31,32,33] and literature cited therein) may have resulted in regional introgression of mtDNA lineages into wild boars at different stages of pig domestication, particularly in southeastern Anatolia. Notably, “pigs” appear already as subject matter in lexical lists of the Uruk period (3900/3700-3000/2900 BC–before Christ), in the Mesopotamian Chalcolithic (before the Bronze age) and in the Early Dynastic period, in addition to “cattle”, “fish”, “bird”, “meat”, and “wool”/”cloth” [34]. Therefore, current lineages among Anatolian wild boars may also represent those of pigs at various stages of domestication. Indeed, a first survey of external phenotypes together with mtDNA lineages of Anatolian wild boars have revealed signals of pig introgression at least according to some suspicious external phenotypes [35]. Early Anatolian pig lineages have been found with only a few exceptions in European pigs; the latter rather harbor typical European wild boar lineages (E1—European lineage system 1, E2—European lineage system 2) due to (prehistoric) introgression of European wild boars. Obviously, an almost complete replacement of all the original Anatolian lineages that have been brought by the Anatolian settlers during the Neolithic period to Europe has occurred [36], and this has been confirmed for the nuclear genome as well [37]. This circumstance offers the chance to discover typical European pig lineages (E1, E2) among Anatolian wild boars, which could stem from prehistoric or historical translocations of European pigs (or wild boars) and subsequent introgression as has been observed in wild boars from the southern Levant [38]. Introgressions of such pig/wild boar lineages from Europe could also have happened during the Ancient Greek and Roman periods in the Middle East [39], the Byzantine Empire (395—1453 A.D.—Anno Domini) [40,41], or even during the period of the Christian crusader states [42], i.e., the “Outremere period”, when the County of Edessa (1098–1144), the Principality of Antioch (1098–1268), the County of Tripoli (1102–1289), and the Kingdom of Jerusalem (1099–1291) were populated by Christians, before the beginning of the Muslim/Ottoman periods during which supposedly pig husbandry was abandoned. A distinction between possible European pig and wild boar lineages in Anatolian wild boars is probably not possible, because translocations and releases of wild boars (e.g., as piglets) cannot be excluded since prehistoric times, as known, e.g., from Cyprus [30,43,44]. Domestic pigs, wild boars, or their hybrids of different generation may also have been imported to Anatolia for instance from Italy at medieval times, as merchants from Venice and Genova (in northern Italy), as well as Pisa (in central Italy), continuously delivered goods to the Christian settlements in Outremere [42,45]. Medieval Italian seafarers also established trade between Cyprus and Outremere and pigs were abundant and consumed in the crusader states, along with goats and cattle [42,45]. Even Far Eastern (Chinese) pig haplotypes in Anatolian wild boars could, in principle, originate from modern European pig breeds that were introgressed by Far Eastern mtDNA and imported to the adjacent regions, such as Georgia and Armenia, especially during the former USSR [46,47,48,49]. In principle they could even originate from Chinese pigs that were possibly brought to the Middle East by medieval merchants along the Silk Road, especially in the 13th century, during the Mongolian period, when overland travel was safer than before [42], e.g., via Turkmenistan and along the Silk Road south of the Caspian Sea to Trebizond (present-day Trabzon in northeastern Türkiye).

Given all the possible scenarios of introgression mentioned above, as well as natural and anthropogenic gene flow, our aim was to investigate the diversity and spatial distribution of mtDNA lineages of wild boars in Türkiye, with a focus on the Anatolian Peninsula by analyzing new samples in combination with that studied earlier from the Middle East [35] and other downloaded sequences. Specifically, we focused on the following questions:

- Can we identify typical ancestral Anatolian lineages, do they have a widespread or limited distribution, and are there even endemic lineages on the Anatolian Peninsula?
- Are there lineages that occur only occasionally but are widely distributed in Anatolia and that occur primarily in other parts of the Middle East, which could indicate gene flow over longer periods of time?
- What is the spatial distribution of typical European (E1, E2) lineages on the Anatolian Peninsula found previously [35], and does their spatial distribution suggest natural gene flow from Europe across the late Pleistocene land bridge, which may have formed a natural migration corridor before its disintegration in the middle Holocene?
- Or do typical European lineages rather indicate historical anthropogenic gene flow (from pigs or wild boars), i.e., translocations and subsequent introgression into local Anatolian wild boars?
- Are there any Far Eastern (Chinese) lineages present in Anatolia that may indicate either relatively recent translocations of European pigs or even import by medieval merchants from Central and Far East Asia?
- Do wild boars on the Anatolian Peninsula have higher mtDNA diversity than wild boars from the European side in the SE (southeastern) Balkans, as, e.g., brown hares, which have naturally expanded their range from the Anatolian Peninsula to southeastern Europe during the late Pleistocene and possibly later in the early Holocene? And are lineages in European Turkish Thrace indicative of evolutionary descendance from Anatolia?

## 2. Materials and Methods

**Figure 1:**
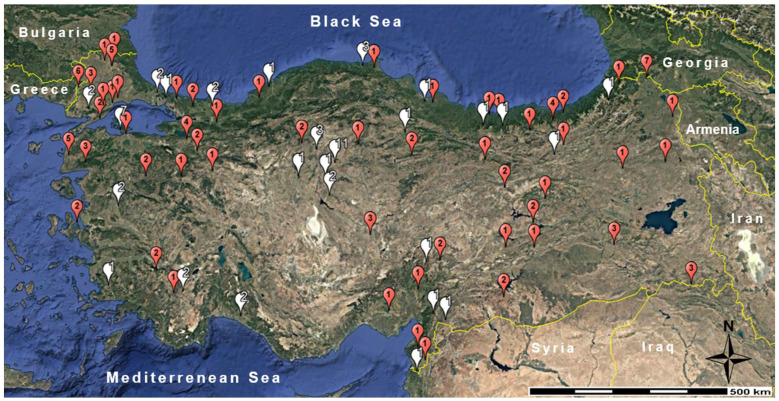
Geographic distribution of 156 *Sus scrofa* samples obtained from 75 different localities in Türkiye. White balloons represent the sampling localities of our earlier study [35], and red balloons represent the localities of the samples obtained in this study. Numbers on the balloons indicate sample sizes.

In this study, both tissue samples collected earlier [35] and newly collected tissue samples were used. Altogether, samples of 156 wild boars, *Sus scrofa*, were collected during regular hunts between September 2013 and February 2024 in 75 different sampling areas across Türkiye (Figure 1, Table S1). No animals were killed for the purposes of this study, and all procedures contributing to this study were carried out within the scope of research permits obtained from the Turkish Ministry of Agriculture and Forestry, Directorate of Nature Conservation and National Parks (KKU BAP Project No: 2014/35, 2018/020, 2021/077). Samples were either stored in 96% ethanol or kept frozen at −20 °C until DNA extraction.

### 2.1. DNA Extraction, Amplification, and Sequencing

Genomic DNA isolation from tissue samples was performed according to the GeneMATRIX Tissue & Bacterial DNA Purification Kit (EURx Ltd., Gdansk, Poland) protocol. A 522 bp fragment of the mtDNA control (D-loop) region of all samples was amplified using the primers 5′-GGAGACTAACTCCGCCATCA-3′ (forward) and 5′-TGGGCGATTTTAGGTGAGATGGT-3′ (reverse) [50]. For details of the PCR see [35]. The PCR products were checked using electrophoresis on 1.2% agarose gels and purified by using ExoSAP (Fermentas, Waltham, MA, USA) following the manufacturer’s recommendations. Sequencing was conducted on an ABI 3100 Genetic Analyzer (Applied Biosystems, Waltham, MA, USA).

### 2.2. Data Analysis

The sequences of all samples were edited using BioEdit v.7.2.5 [51]. A multiple alignment of the sequences was performed using the Muscle algorithm implemented in MEGA X [52], and final adjustments were performed by eye. All new sequences generated in this study, as well as the sequences obtained earlier [35], are deposited in GenBank (accession numbers PV600821-PV600835) (Supplementary Table S1).

We used two data sets for our phylogenetic and spatial genetic analyses. The phylogenetic analyses were performed with an additional 1690 domestic pig and wild boar sequences downloaded from GenBank (S1) to estimate the phylogenetic relationships among wild boars across the entire species range worldwide, as covered by the presently available data. The phylogenetic analyses were carried out on a data set comprising a 376 bp fragment of the first hypervariable segment of the mtDNA control region. Based on the nucleotide differences between sequences, haplotypes were determined in DnaSP v.6.12.03 [53]. The phylogenetic relationships among haplotypes were first constructed with the maximum likelihood (ML) criterion and under the Timura-Nei (TN93)+G+I evolutionary model according to the Corrected Akaike information criterion (AICc) and the Bayesian information criterion (BIC) using MEGA X. The robustness of the trees was determined by bootstrap resampling (1000 replicates) [54]. Secondly, a Bayesian phylogenetic inference (BI) tree was constructed in MrBayes v.3.2.2 [55] and the best-fit model of nucleotide substitution was determined using jModeltest 3.7 [56] according to the Akaike information criterion (AIC). The Bayesian phylogenetic analyses were carried out using the Timura-Nei (TN93)+G+I model of sequence evolution and two independent runs of four Monte Carlo Markov chains (MCMC- one cold and three heated) for 14 million generations, sampling every 1 million generations. The first 25% of the sampling trees and estimated parameters were discarded as burn-in. To produce the phylogenetic tree images, Figtree v.1.4.4 [57] was used. The mitochondrial DNA sequence of *Sus barbatus* (GenBank accession number: AJ314540 [58]) was included as outgroup taxon in both analyses. A median-joining (MJ) network was created using NETWORK v.10.2.0.0 [59] with default specifications for modeling the phylogenetic relationships between the haplotypes by allowing reticulate evolutionary scenarios and alternative pathways.

Based on the ML and BI trees and MJ network, the sequences of the worldwide data set were divided into the following seven mtDNA clades: Near Eastern NE1 and Near Eastern NE2; European E1 and European E2; Clade1, Clade 2; Asian Clade3—in principle according to earlier findings (see below). Descriptive statistics [number of polymorphic sites (S), number of parsimony informative sites (PIS), number of haplotypes (H), haplotype diversity (Hd), nucleotide diversity (π), average number of nucleotide differences among haplotypes (k), Watterson’s estimator theta (per site) (θ_w_)] for each group in the data set were calculated in DnaSP [53]. The population genetic structure between and within the analyzed groups was estimated by analysis of molecular variance (AMOVA), and *F_ST_* statistics [60] were calculated with 10,000 random permutations in ARLEQUIN 3.5 [61]. ARLEQUIN was also used for neutrality analysis as Tajima’s D [62] and Fu’s Fs [63].

As a second approach we performed spatial genetic clustering of the newly obtained sequences from Türkiye and the previously reported earlier sequences [35]; for that analysis we included additional 204 wild boar sequences from regions in the southern Balkans (i.e., North Macedonia, Greece, Bulgaria), as well as Cyprus, Iran, Azerbaijan, and Armenia, downloaded from GenBank (S1). The final alignment of the combined data set for that spatial analysis comprised 371 bp. All analyses were performed on sequences without indels. Specifically, we used Geneland [64] to assess genetic clustering while accounting for the spatial locations (geographic coordinates) of individuals. A correlated allele frequencies model was applied in 5 runs, with 1 million MCMC iterations, sampling every 100 steps, and the number of clusters (K) ranging from 1 to 10 each. The run with the highest likelihood was used to determine the most probable number of genetic clusters. Subsequently, an additional Geneland run was performed with the same parameters as before, but with K fixed to the most probable number of clusters identified in the initial runs.

## 3. Results

All new samples yielded 522 bp segments of the mtDNA control region (D-Loop) of 103 wild boar individuals from almost all regions of Türkiye, especially from NW Anatolia, Turkish Thrace, Central Anatolia, E Anatolia, and NE Anatolia. The D-Loop sequences of 53 individuals produced by the same authors [35] were also included in the present data set. Together with those sequences and an additional 17 Turkish wild boar sequences downloaded from GenBank (n = 1 [65], n = 2 [66] and n =14 [67]), a total of 173 sequences represented 15 haplotypes (H1–H15, see Supplementary Table S1). Seven of these haplotypes (H2, H6, H8, H10, H12, H13, and H14) were new haplotypes, i.e., they have so far not been reported in the wild boar. Among all 1846 D-Loop sequences analyzed presently (i.e., including all downloaded ones from GenBank), 202 haplotypes (H1–H202) were identified with 93 variable sites and 66 parsimony-informative sites. In this context it should be mentioned that all haplotype numbers we report hereafter refer only to the present study and not to any previously published haplotype numbers/labels, since previously published D-loop alignments vary somewhat in the literature and are not identical to our present alignment lengths. Overall and clade-specific values of variability (i.e., Hd, π, k, and θ_w_) are given in Table 1.

**Figure 2:**
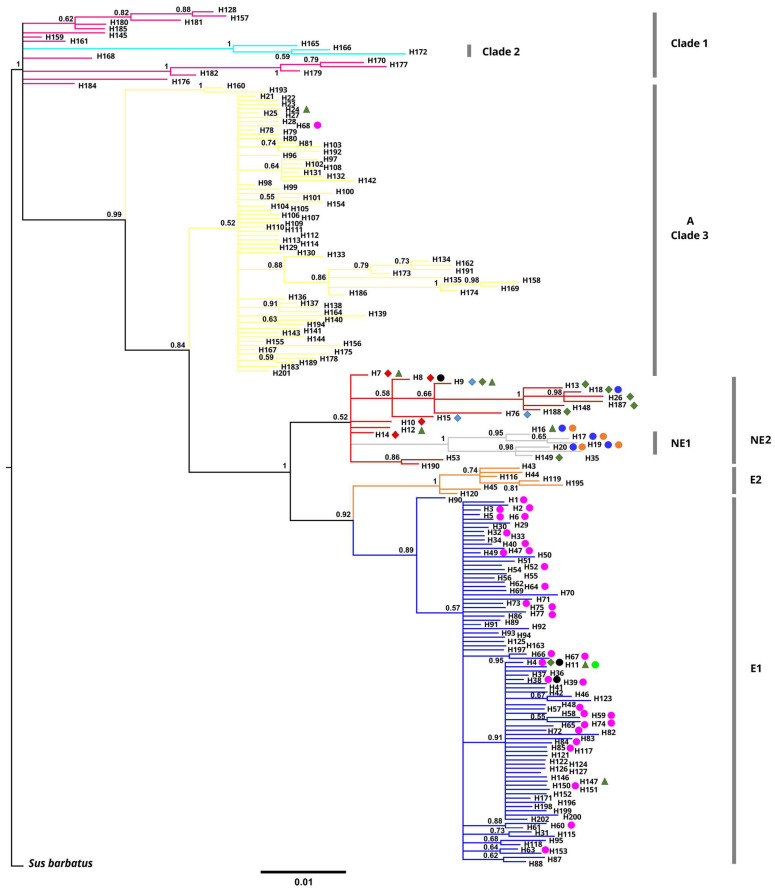
BI phylogenetic tree. Haplotype codes correspond to the ones mentioned in the text and given in Supplementary Table S1. Symbols with haplotype codes correspond to the symbols in Figure 4 according to our spatial clusters obtained from the Geneland analysis.

**Figure 3:**
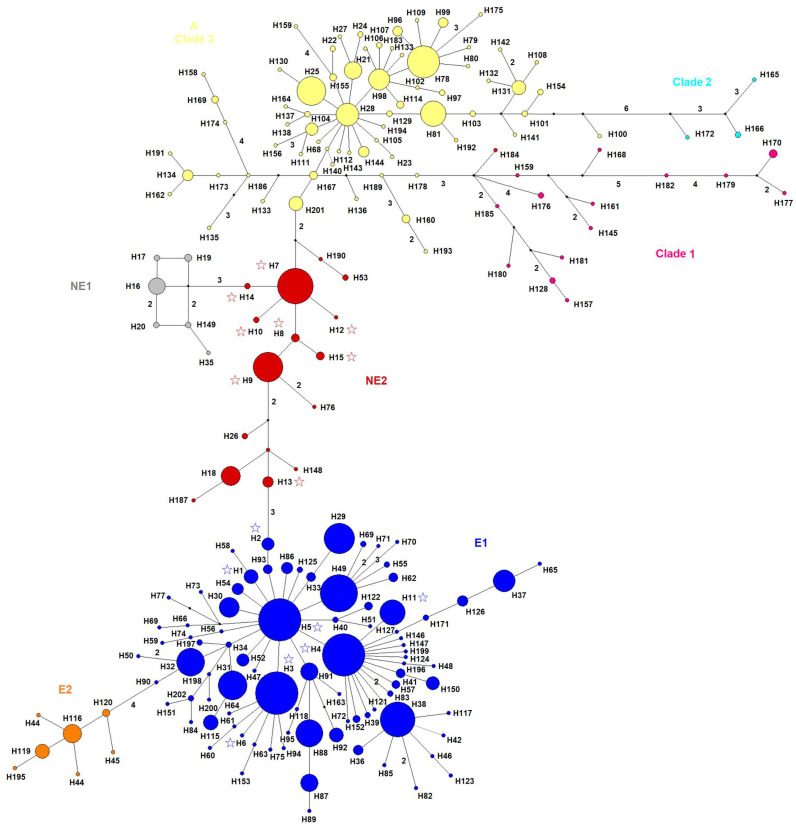
Median joining network of D-loop haplotypes of the presently studied wild boars, including all downloaded from GenBank. Stars denote haplotypes from Türkiye, i.e., the new ones and the ones found earlier [35]. Circles present haplotypes and their phylogenetic position, with circle sizes being proportional to haplotype frequencies. Numbers along connecting line indicate numbers of substitutions, if more than 1.

Both our tree-generating phylogenetic analyses (ML tree in Figure S1 and BI tree in Figure 2) and our MJ network (Figure 3) grouped all wild boar sequences into seven major clades or phylogenetic groups, as already reported earlier ([35] and literature cited therein [68]): three Asian clades/haplogroups (Clade 1, Clade 2, Asian Clade 3), Near East 1 (NE1), Near East 2 (NE2), Europe 1 (E1), and Europe 2 (E2, Italian clade). However, support for individual clades was partly relatively low, particularly in the ML model, and there were no big phylogenetic breaks in the network compared to the evolutionary divergence within the clades/haplogroups. Nevertheless, our AMOVA indicated that about three quarters of the total observed relative genetic variability were partitioned due to those seven phylogenetic groups/clades/haplogroups (Table 2).

The E1 clade and all three Asian clades exhibited a high degree of genetic diversity (Table 1). In both phylogenetic trees, haplotypes that were found in Türkiye were grouped almost equally to the E1 (H1–H6, H11) and the NE2 (H7–H10, H12–H15) clades, and this was confirmed by the MJ network. Remarkably, the European H4 haplotype was observed in wild boars from the Kırklareli province in the Turkish Thrace region (i.e., the southeastern Balkans), but also on the Anatolian side of Istanbul, and in the Gaziantep and Hatay provinces in SE Anatolia. The H11 haplotype was observed in wild boars from NE Anatolia and in the Şanlıurfa province in SE Anatolia, even though that haplotype clustered into the E1 haplogroup.

Two Turkish haplotypes (H1–H2) of the E1 haplogroup were currently revealed only at relatively low frequencies in Turkish Thrace of the SE Balkans; while H1 was found already earlier in various parts of the Balkans at a very low frequency, H2 was recovered for the first time in our samples only from Turkish Thrace. In contrast, the other four haplotypes (H3–H5, H11) of the E1 haplogroup were found at relatively high frequencies. The relatively derived haplotype H6 of the E1 haplogroup was found in only one wild boar individual from the European side of the Istanbul metropolitan area. The two most frequent and widespread Turkish haplotypes H7 and H9 were found at relatively basal evolutionary positions of the NE2 clade (Figure 3), i.e., closely related to and derived from the Asian clade 3; they were both distributed in W Anatolia, also close to the southern coastline of the Sea of Marmara, i.e., the region of the late Pleistocene/early Holocene land bridge to southeastern Europe, across the Anatolian Peninsula to S and SE Anatolia. H9 was also found in Iran. Haplotype H7 formed the basis of several derived haplotypes (H8, H10, H12, H14, H15) and linked via H14 to the NE1 haplogroup, which appeared merely as a little tightly related side system of evolutionary lineages of the more basal NE2 clade (Figure 3).

One evolutionarily modern Turkish haplotype (H13) within the NE2 clade and the Turkish haplotype H2 within the major Europe clade/haplogroup E1 connected the Near Eastern and the European lineages (Figure 3). Of these two haplotypes, H2 has so far been found only in Arnavutköy in the European part of Istanbul and in Edirne in the SE Balkans, while somewhat surprisingly H13 has only been found at different localities in SE Anatolia (i.e., the Hakkari, Batman, Kilis, and Hatay provinces). According to our network, the E1 haplogroup is separated from the NE2 haplogroup by only three mutations, and the NE2 haplogroup is separated from the Asian haplogroup 3 also by only three mutations.

**Figure 4:**
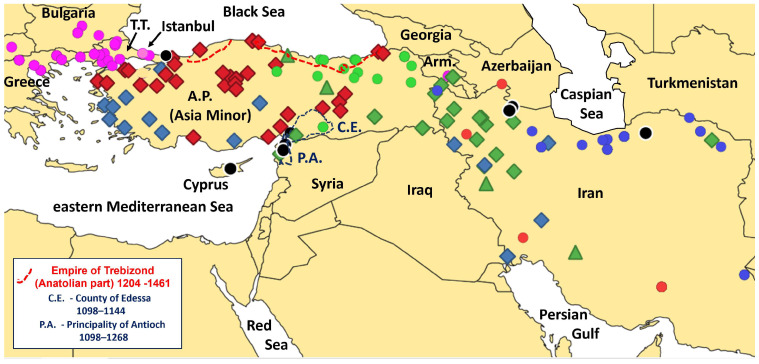
Spatial distribution of individual mitochondrial haplotypes and their assignment to one of the eight clusters (symbols) according to the Geneland results. The symbols correspond to those shown in the phylogenetic tree in Figure 2. The single light pink circle indicates an individual from the European side of Istanbul and the pink circle right to it indicates an individual with an E1 clade haplotype from the Asiatic side of Istanbul. C.E. and the associated dashed line indicate the territory of the medieval County of Edessa (1098–1144 A.D.), P.A. that of the medieval Principality of Antioch (1098–1268 A.D.), and the red dashed lines indicate the Anatolian part of the medieval Empire of Trebizond (1204–1461 A.D.). A.P.—Anatolian Peninsula, T.T.—Turkish Thrace in the SE Balkans, Arm.—Armenia.

Our Geneland model yielded K = 8 genetic clusters inherent to the considered 360 sequences from Türkiye, Iran, Armenia, Cyprus, and the Balkans (from Greece, Bulgaria, and North Macedonia) (Figure 4). However, ten (0.278%) individual sequences could not be assigned convincingly (i.e., with < 90% probability) to one of the eight genetic clusters. All the other 350 sequences were clearly assigned to one of the eight genetic clusters as indicated by our Geneland results with an average probability of 0.9996% and a minimum probability of 0.9732%. Two of the ten sequences that could not be assigned with high enough probability (i.e., black circles in Figure 4) were from two wild boars from Kandıra in NW Anatolia with haplotype H8 of the NE2 clade, a sequence of expected geographical and phylogenetic positions. The remaining eight sequences that could not be assigned properly to one of the eight Geneland clusters encompassed one wild boar from Islahiye (S Anatolia), one from Hatay (S Anatolia), one from Altınözü (S Anatolia), one from Nemin (Ardabil, Iran), one from Sabalan (Ardabil, Iran), one from 16 km east–southeast of Gorgan (Golestan, Iran), and two from Cyprus (Figure 4). All those latter individuals carried haplotype H4 (E1 clade), except for one, which was from Cyprus and carried haplotype H38 (E1 clade).

## 4. Discussion

In our first report on mitochondrial lineage diversity in wild boars from Türkiye [35], we found considerable variability but concluded that a comprehensive phylogeographic interpretation of the spatial pattern of our D-Loop sequences must be based on a larger set of samples and a wider geographical coverage. This also referred to the interpretation of the haplotypes encountered on the Anatolian Peninsula that showed typical European lineage characteristics, i.e., that clustered phylogenetically with the major European wild boar phylogroup (E1, according to [35] and literature cited therein, [50]). In the present study we have added samples of 55 (i.e., 21.1% more) new locations from various bioclimatic regions in Türkiye, particularly from SE and E Anatolia, the ancient center of pig domestication in the western Palearctic [69] and part of the suggested meeting zone of the Near East 1 (NE1) clade and Near East 2 (NE2) clade of D-Loop lineages [70] (Figure 1). However, we could not support such a meeting zone for haplotypes of the NE1 and NE2 clades by our results based on the presently used samples.

### 4.1. Turkish Wild Boar Subspecies and Domestication

Some distribution maps of the wild boar for Türkiye suggest an absence of this species from parts of Turkish Thrace, the European part west of the Bosphorus/Sea of Marmara/Strait of Çanakkale waterway between the SE Balkans and NW Anatolia [28,71], whereas others [9,72,73] suggest that wild boars are distributed throughout Turkish Thrace and across entire Anatolia. A continuous distribution range in entire Türkiye (including Turkish Thrace) is stated for both *Sus s. scrofa* L., 1758 (Yabani domuz) and *Sus s. libycus* Gray, 1868 (Evcil domuzun atası), with the latter form (subspecies) supposedly representing the primary ancestral basis of all initial domestic pigs in the western part of the Palearctic [9]. The latter form, with reddish brown coat color, is not common in Anatolia; rather it is mainly found in the Mediterranean regions [9]. However, since this form (subspecies) has only been described based on phenotypic characteristics, it is questionable to what extent it represents pure wild boars or individuals variably introgressed by ancestral and/or more modern domestic pig gene pool elements.

The most recent checklist of mammals from Türkiye lists *Sus s. scrofa* L., 1758 and *Sus s. libycus* Gray, 1868 with the type locality of the latter being “Xanthus”, i.e., the ancient Greek settlement of Ξάνθος, near “Günek” (which we could not identify on the map) in SW Turkey close to Muğla [9,10]. Taxonomically, we like to note that the above form *Sus scrofa libycus* Gray, 1868 was originally described as *Sus libycus* by Gray 1868, as synonym to *Sus scrofa* L., 1758, but later by Groves as *Sus lybicus* (sic!) [74]. Following the current taxonomic rules, the correct subspecies name is *Sus scrofa libycus* ([74], see, e.g., also “GBIF Backbone Taxonomy”, https://www.gbif.org/species/4262891, accessed on 27 January 2025). Its current distribution in Türkiye is not known in detail, due to the absence of fine score geographic data of both phenotypes with a full coverage of Anatolia. Specimens from the Kırıkkale region in north-central Anatolia were considered *Sus s. scrofa* [75], following the classification criteria of Genov [76], who considered only four subspecies of wild boar to occur globally and who integrated *Sus scrofa lybicus* [sic!] into *Sus s. scrofa*, with the latter subspecies occurring basically in Europe (and further east) and in the Middle East. That latter view was in contrast with the earlier position that wild boars from the Anatolian Peninsula represented all *Sus s. libycus* [77]. Four wild boar specimens from the Kırıkkale region in central Anatolia showed a diploid chromosome number of 2n = 38 and a number of autosomal arms NF-a = 60, identical with that of domestic pigs and of several wild boar specimens from several regions in central Europe and the Balkans with diploid chromosome numbers between 36 and 38 [78].

That latter karyological data, together with the earlier found external phenotypes and typical E1-lineages among our earlier D-loop sequences [35], may suggest the presence of introgressed domestic pig lineages in wild boars roaming perhaps many regions of the Anatolian Peninsula, and *S. scrofa libycus* could simply represent individuals with variable levels of introgression of pig-typical genetic characteristics. Unfortunately, we did not have external phenotype information at our disposal for all currently studied samples. But remarkably, the two haplotypes H9 and H15 (blue diamonds in Figure 4) from SW and W Anatolia (including a location close to Muğla), the region of the type locality of *Sus scrofa libycus*, represent relatively basal NE2 lineages, rather than E1 or E2 lineages (that include modern pig breeds). Notably, our results suggest that one (H15) of these latter two Anatolian haplotypes is endemic to the Anatolian Peninsula, whereas the other one (H9) is widespread in the Middle East including Iran [79]. Haplotype H9 is also present on the Greek island of Samos off the coast of SW Anatolia [79,80]. It could have reached that island either naturally by individuals that have swum across the short distance of ca. 2 km from mainland Anatolia (Strait of Mycale) or already by natural immigration over the late Pleistocene land bridge with mainland Anatolia, as suggested by the 50 m isobath line that connects the island with mainland Anatolia ([81], see also [82]); alternatively, but to our understanding less probably, wild boars (e.g., as piglets) or ancient pigs could have been translocated by ancient or more modern settlers from Anatolia to Samos later in the Holocene. As to our knowledge, the currently found haplotype H9 as well as the earlier found NE2 haplotype H76 from Samos have not been reported from anywhere else in Greece or other regions of Europe [79,80,83], which is in line with our conclusion about their Anatolian origin on Samos. Remarkably, however, microsatellite data of wild boars from Samos indicated introgression by pigs or wild boars of mainland Greek origin ([80], fig. 2B). The currently revealed absence of European E1 haplotypes in wild boars from the SW Anatolian mainland suggests no or very little (anthropogenic) ancient or historic gene flow from Samos to mainland Anatolia. In any case, the two phylogenetically closely related NE2 haplotypes H9 and H76 of wild boars from Samos indicate their evolutionary connection to Anatolian (Middle Eastern) lineages [80,83]. Future studies, ideally involving historical and prehistorical samples, may link these three Anatolian haplotypes also with (ancient) domestic pigs from eastern Asia Minor and support our hypothesis that *Sus scrofa libycus* (phenotypes) represent merely individuals of *Sus s. scrofa* with variably introgressed portions of (ancient) domestic pig elements. The currently observed predominant distribution of two of these lineages in wild boars from W and SW Anatolia, rather than in lineages from the supposed region where pig domestication has started in E and SE Anatolia—e.g., ([84,85], see also [86] for sociological interpretations of wild boar and other animal statues in the Göbekli Tepe Neolithic Cultural Region), may be due to anthropogenic translocations somewhat later in the Anatolian domestication history of pigs: in fact, long-distance trade has already been inferred for local cultures of ancient eastern Anatolia, such as from eastern Anatolian Hallan Çemi in the 11th Millenium BP, where both wild boars and pigs were identified among the food remains [84]. Later on in the history of the many Anatolian peoples [87], large-scale mobility over land (and sea) has affected the formation of regional cultural identities, particularly among peoples in the first half of the Iron Age (1200–600 B.C.), and this has led to different pig husbandry practices in Anatolia with supposedly more extensive pig breeding and possibly a higher chance of introgression (specifically of female lineages) into wild boars in W Anatolia [88]. In any case, both the haplotypes H9 and H76 are relatively modern lineages of the NE2 clade, which accords to the earlier conclusion from archeological and genetic data that the primary Anatolian pig domestication has not involved NE1 clade lineages [67]. The absence of NE1 haplotypes from all currently studied Anatolian wild boars, especially from those of eastern, central-eastern, and southeastern Anatolia, but their presence together with NE2 haplotypes in southern (and western) Iran and other regions of the Middle East as well as in (historic) Egypt and the Northern Caucasus region ([67], fig. 2 and suppl. fig. S2b, suppl. material online, [68,70], see also suppl. tab. S1), is consistent with the previous conclusion that the domestication of pigs in western Eurasia began in E and SE Anatolia.

The geographical distribution of our samples suggests a largely continuous range of wild boars in Türkiye, except for small areas particularly at high altitude in mountainous regions and steppe plains with little vegetation, where habitats and climate are too harsh and too unfavorable for wild boars, such as in a small area of SE Anatolia. Remarkably, wild boars also occur in E Anatolia (e.g., Erzurum and Kars regions), which is known for its harsh winter climate, but also in S and SE Anatolia (e.g., Şanlıurfa region) with hot and dry summer climate [6]. A very recent distribution map based on a systematic country-wide survey is, however, not available. Population densities throughout entire Türkiye seem to be on average comparatively low, as suggested by modeling results [72]. The latter study infers relatively high population densities in the west, northwest, and north of the Anatolian Peninsula and along the seaboard of the Black Sea in Turkish Thrace, reaching ca. one head per square km, and low population densities in central, eastern central, and E Anatolia towards the borders with Armenia, Azerbaijan, Iran, Iraq, and Syria, reaching a level of 0.1 heads per square km. For SE Anatolia no modeling results are available, but the same study [72] may suggest that wild boars are absent from most parts of Syria and NW Iraq. Hence, potential gene flow into our Anatolian study region could have occurred over many generations and in recent times most likely only from the southern Caucasus region (i.e., Georgia, Armenia, Azerbaijan), NW Iran, NE Iraq, and NW Syria, i.e., the Levant along the Mediterranean seaboard.

### 4.2. Gene Flow Across the Bosphorus/Sea of Marmara/Dardanelles Waterbody

In the northwest of the Anatolian Peninsula, gene flow could have occurred across the late Pleistocene land bridge with the SE Balkans [82] and, in principle, also later on and still today, given that wild boars are fairly good swimmers [89] and should be capable of crossing the Bosphorus and the Strait of Çanakkale, which are not much wider than 1 km at the narrowest section (albeit probably with fairly strong currents, particularly in the Bosphorus). Moreover, these days three bridges over the Bosphorus may also provide occasional migration. Given those historical geological and current geomorphological scenarios for NW Anatolia and the SE Balkans, it is remarkable that our findings (Geneland model) based on a numerically and spatially representative sample suggested a large absence of historical and current (maternal) gene flow in both directions: in fact, we found only one wild boar with a typical E1-clade haplotype of the Balkans and specifically of Turkish Thrace, currently in NW Anatolia, not too far away from the Bosphorus (Figure 4). It may indicate occasional natural gene flow or historic anthropogenic translocations from Europe, for example, during Byzantine times (when transport of pigs was also carried out by ship along the coast to support the markets of Constantinople—see below).

At the same time, the absence of typical Anatolian haplotypes of the NE2 clade in the wild boars from Turkish Thrace currently studied is compatible with the assumption that no (natural) gene flow from the Anatolian Peninsula to the southeastern or southern Balkans has occurred [35,80]. However, it contradicts a recent spatial model of D-loop lineages that suggested mitochondrial gene flow in wild boars from Anatolia to parts of Turkish Thrace, but without support from any samples ([67], fig. 2a and suppl. data). The latter model did not expect E1 haplotypes to be found in NW Anatolia either, which is broadly consistent with our present findings. However, European E1 lineages were found earlier in modern wild boar samples (TP1, WBTR514) from western Anatolia, in addition to E1 lineages in other parts of the Middle East as well as in domestic pigs from various ancient excavation cites in Anatolia ([67], suppl. tab. S2). Those findings were considered to indicate ancient (prehistoric) anthropogenic translocations of European wild boars and/or pigs to Anatolia. Under the assumption that these ancient pig lineages had sufficient time to introgress over many generations into Anatolian wild boars, they could have been expected—at least occasionally and locally—in Anatolia, or even spread across regions, given the long time for potential dispersion ([67], suppl. tab. S2). Indeed, DNA evidence and dental morphometry of archeological samples suggested that pigs with E1 lineages were imported to Anatolia at the latest during the Middle to Late Bronze Age [67]. However, such lineages in Anatolia and other regions of the Middle East could, in principle, also originate from later anthropogenic translocations of pigs (or wild boars?) in the Hellenic–Roman period, or later on in the Byzantine period, during the European Crusades, the European colonial period in the Crusader States in Anatolia and the Levant, or during the period of the independent empire of Trebizond in N and NE Anatolia. Furthermore, recent natural immigration of wild boars with introgressed European (E1 and E2?) or even Asian (Far Eastern) mtDNA may be expected from the former USSR republics northeast and east of Anatolia, i.e., from Georgia and Armenia, which have had Christian populations with partly extensive pig husbandry until today [47,48,49,90]. However, whereas Far Eastern Asian lineages were currently not found in (NE) Anatolia, several Asian haplotypes were observed in wild boars from Iran and Turkmenistan, particularly from north of the Elburz Mountain range more or less along the southern seaboard of the Caspian Sea [36,68,70], in the region along the medieval Silk Road. In addition to a plausible natural range expansion of wild boars from Central Asia, it may suggest historic introgression by pigs traded by medieval merchants from more eastern, particularly Central Asiatic, regions [66,70] (see Supplementary Table S1 for respective accession numbers and associated haplotypes of our current numbering). Indeed, pig farming was practiced, for example, already by the Anau and the Khust cultures, ancient agricultural civilizations of Central Asia, with the former centered in S Turkmenistan and the latter—since the late Bronze Age and the early Iron Age—in the Fergana Valley of eastern Uzbekistan that connects to Kyrgyzstan and eventually to western China [91]. In any case, our present study suggests that both a potential natural range expansion and historical pig trading along the Silk Road and subsequent introgression into wild boars may not have reached Anatolia (even though Trebizond was one of the major medieval trading posts of the Silk Road).

### 4.3. Pig Farming in Prehistorical and Historical Times in Anatolia and Signals of İntrogression of Pigs into Modern Wild Boars from Anatolia

In prehistorical and historical Asia Minor, the Levant, and Cyprus both wild boars and pigs were used as food by local civilizations throughout long periods of time, such as the Neolithic civilizations of Cayönü and Göbekli Tepe in SE Anatolia [87], and translocations of both wild boars and pigs were very likely [30,31,84,88,92,93,94,95,96]. From the pre-Roman Hellenic period a historic document is known that hints towards wild boar hunting in addition to zooarchaeological data: specifically, under Persian administration, tribute had to be paid to the Satraps or other administrative authorities, and “…one leg each from the hunted wild boar and red deer” had to be delivered, among other (agricultural) goods (cf. [87] p. 217). Long-term gene flow from wild boars to pigs with different degrees of domestication could have occurred over many generations, as suggested for instance by intermediate tooth sizes in excavations of prehistoric cultures [31,84]. However, it is unclear to what extent natural gene flow from pigs to Anatolian wild boars could have also occurred, and to what extent introgressed domestic lineages may have survived until today in wild boars. Naturally, only the introgression of mtDNA of European and Far Eastern pig origin would have left distinct mitochondrial signatures in the currently studied Anatolian wild boars, while archaic Anatolian lineages of pigs cannot be distinguished from indigenous wild boar-typical lineages. In the Archaic and Hellenic–Roman periods, pig breeding was practiced both for food production and for religious reasons; pigs were sacrificed to the deities alongside other animals (such as sheep, goats, and chicken)—pigs especially were sacrificed to Demeter, the Olympic goddess of fertility, agriculture, harvest, and seeds [97,98]. During the Roman and Byzantine periods, pig farming was widely practiced in Anatolia and wild boars were present in big quantity and hunted [40,41] and [87] (p. 504): a Roman sarcophagus, probably from the 4th century B.C., depicting the hunt of the grave owner with spear on horseback against a male wild boar caught by dogs ([87], p. 223), may be indicative of popular wild boar hunting in those days. Equally, extensive pig husbandry may have been widespread as well, as suggested, for example, by a report on a dispute between neighboring farmers from the time of the Roman emperors concerning the hiding of pigs that had strayed from the neighboring property ([87], p. 576). Notably, both pigs and wild boars were valued as food by the Romans, as the manual of “Trimalchio’s Dinner Party” in the novel “Satyricon” by T. Petronius Arbiter (14–66 A.D.) may suggest, which lists both wild boar in “cena–second course” and white pigs in “cena–third course” (cf., [99]). During the Byzantine period pigs were probably less common than sheep and goats among the animal food of the people from the countryside [40], but the regulation of pork butchers in the “Book of Eparch” [41] (p. 103) suggests nevertheless quite some importance (especially for the military), and both intensive and extensive pig farming could have been practiced in various regions of Asia Minor, such as Cappadocia [41]. In the “Geoponica 19.6” it is also mentioned that the pigs needed protection from the cold weather in styes over winter, but in the warm season they were allowed to feed in the forest (e.g., on *Quercus zerris*) cf. [41]. Pigs were also led by swineherds over longer distances or transported by ship along the coast of the Mediterranean, the Black Sea, and the Sea of Marmara, and a report about a private land owner, who mainly owned properties in the European Balkans (Strymon valley), and who claimed to have lost “1000 pairs of oxen, 50,000 pigs and 70,000 sheep in the course of the civil war of 1341–1354 A.D.” (likely an exaggeration), may indicate that pork was an important food for the Byzantine population, produced either in large-scale breeding units (especially also for the army) or by many small family farms for subsistence [100,101,102].

Corresponding to the many generations of potential introgressive hybridization of pigs imported from Europe since prehistoric times into the presently studied wild boars, we found many E1 lineages in the Anatolian samples; altogether, 26.4% carried an E1 haplotype—remarkably only two different, either H4 or H11. These two haplotypes of European origin were not evenly distributed across our study region; rather, we identified two spatial clusters of E1 haplotypes, a small one in S Anatolia and a large one in NE Anatolia (black and green circles, respectively, in Figure 4). In S Anatolia both H4 and H11 were found, while in NE Anatolia only H11 was present. H4 is widely distributed among wild boars from continental Europe, including Italy, France, the Iberian Peninsula, Germany, the Balkans, and parts of Eastern Europe, as well as other regions like Britain, Sweden, Sardinia, Corsica, and Cyprus (Supplementary Table S1). It was also found in many modern domestic pig breeds from continental Europe and Britain and even in feral pigs of New Zealand. It holds a central phylogenetic position that forms the basis of a starlike radiation. In Türkiye it occurs at a rather low frequency in Turkish Thrace on the European side of the Bosphorus/Sea of Marmara/Dardanelles water barrier to Anatolia, most probably due to its wide (natural) distribution in the Balkans; according to the data downloaded from GenBank and the associated literature, its nearest European occurrence was reported from Pella, N Greece [79,80]. The single wild boar with the H4 haplotype on the Anatolian side of the Bosphorus/Sea of Marmara/Dardanelles migration barrier could have descended from (historical) anthropogenic translocations of pigs or wild boars in Byzantine times or earlier, or ancestral female wild boars could have reached NW Anatolia by swimming across the Bosphorus. However, the restricted spatial signal for the H4 and H11 haplotypes in S Anatolia rather may suggest anthropogenic introductions. In fact, it corresponds very well to the region of two of the four medieval Crusader states in the Middle East, namely the County of Edessa (1098–1144 A.D.) and the Principality of Antioch (1098–1268 A.D.) and may thus indicate a historical introgression of pigs imported by the European settlers of the Crusader states. Given the current and historical presence of wild boars along the seaboard of the Levant [93,95,102], they may also have originated from gene flow over generations from the southern Levant, where the two other Crusader states, namely the County of Tripoli (1102–1289 A.D.) and the Kingdom of Jerusalem (1099–1291 A.D.) existed, and where pigs were common food as well [38,40,92,95,102]. Equally, they could have originated from earlier (prehistorical) imports into the Middle East, because our H4 and H11 sequences also matched the alignments of two haplotypes from “NE Armenia” (access. nr. JX894158), and Turkey (“Tp1”, access. nr. JX894183) reported for ancient/historical samples [66,67], and H4 matched sequences published earlier from NW (access. nr. KR075769, KR075770) and NE (Gl789; access. nr DQ872954) Iran and Iraq (GL779; access. nr. DQ872946), and it was previously also found in Cyprus (Gl1041; DQ872982) together with H38 that is also a common E1 haplotype in (south) Europe and in pig breeds [66], but which we did not find among our presently studied samples from Anatolia. The uncertain assignment to the genetic clusters of all these E1 haplotypes (i.e., black circles in Figure 4) in our Geneland analyses was probably due to difficulties of the underlying algorithm to deal with their widespread geographical distribution in our combined data set.

During the Crusade period pig farming was common in Europe, which may have prompted the settlers in “Outremere”, i.e., the Crusader states, to import pigs from their earlier homelands, specifically, Italy, France, and Germany. In fact, our haplotype H11 was also identical with the alignments of sequences of the following accession numbers—JX894162 [67], EU 362586, EU 362592, EU362594-96 [103]—labeled as ”Nera Siciliana” in our “NCBI Blast Search” results. “Nera Siciliana”, or the “Nero Siciliano pig” is a breed particularly known from Sicily, Italy, where pig farming continued from the Roman to the Byzantine times as well as under Norman and Aragonese rules, with a minor interruption during the Islamic period (9th–11th century A.D.) particularly in rural settlements [104]. Notably, the Roman-German emperor Frederik II (1194–1250 A.D.), Federico II di Svevia, was born in Italy and reigned over large parts of today’s Italy, including Sicily (the “Kingdom of Sicily”) from 1198 A.D. onward. He held the title “King of Jerusalem” through his marriage (*iure uxoris*) with Isabella II, the Queen of Jerusalem, and participated in the crusade 1228–1229. His grandfather the German-Roman Emperor Frederic I, Barbarossa, participated in the third crusade [42] but died on his way to the “Holy Land” in Anatolia. Earlier in Europe he received 16,590 pigs plus 2802 piglets annually among other goods for his entourage that encompassed over 1000 people (notably, since the Salian Emperor Otto III, i.e., from ca. 1000 A.D. onward, Roman-German emperors traditionally possessed over 100,000 pigs) [105], but we could not find any information if pigs would have been taken on the crusades from Europe to the Near East. At least a very intensive trading was established especially between Italy (and other regions of Europe) and Outremere as well as Cyprus by Venetian, Genoese, and Pisan merchants and also between Outremere and Cyprus, which was conquered by Richard I (“Lionheart”), king of England, during the first Crusade (1095–1099 A.D.) and was commercially tied to the crusader states [42,106].

Our E1 haplotype H11 (green circles in Figure 4) is by far the most common (73.7%) among all wild boars in NE Anatolia. The two other haplotypes in this region, namely H7, and H12, are NE2 lineages likely endemic to Anatolia, with the former being widely distributed (including previously reported samples [67]) and the latter being singular. Both represent evolutionarily relatively ancestral haplotypes, with H7 occupying a central evolutionary position as basis for to the younger NE1 lineages in the Middle East via H14, which occurs only twice in S Anatolia, and provides a link to the Asian clades (Figure 3). The dominant presence of H11 in NE Anatolia coincides well with the territory of the (Anatolian part of the) Byzantine Empire of Trebizond, which existed after the fourth crusade between 1204 and 1461 A.D. and the Ottoman conquest of Constantinople in 1453 A.D. [42,107]. Notably, Venetian and especially Genoese merchants established important trading posts along the Black Sea coast, such as in Trebizond, Sinope, Simisso (modern Samsun), and Samastri (modern Amasra), and a population of Italian immigrants became fully integrated into the peasantry in rural areas of the (Anatolian part of the) Empire of Trebizond [108,109,110,111,112]. Hence, the import of Italian pigs carrying the H11 haplotype to NE Anatolia by Italian immigrants and its subsequent introgression into wild boars might explain the predominance of this haplotype in NE Anatolia, given the trade of the Empire of Trebizond especially with the adjacent regions to the south and east, including Armenia and Georgia [99,113]. This is highly consistent with the current distribution of H11 in wild boars. Alternatively, it could have been translocated from Outremere by overland trade via the eastern central Anatolian trading hub of Melitene (today’s Malatya), which in medieval times was connected by long-distance roads with both S Anatolia and Trebizond, in addition to other regions in Anatolia [114]. Remarkably, Genoese merchants established a trading post in the NW Iranian Tabriz, which may also help explain the occurrence of the E1 haplotype H4 in NW Iran (and NE Iran and Iraq?) along the Silk Road network, even though particularly camels were traded [113].

### 4.4. Endemic Lineages and Lineages with Wider Middle Eastern Distribution

In addition to the endemic Anatolian haplotypes H7 and H14 mentioned above, five other haplotypes (H8, H10, H12, H13, H15) of our currently sequenced and of our earlier samples [35] were found exclusively in the Anatolian Peninsula; only haplotype H9 showed a wider geographical distribution with additional occurrences in Iran [68] (see above in the text). Altogether, based on the combined data of these two sets of samples (n = 96), 82.3% of all Anatolian wild boars without introgression by European E1 lineages carried endemic NE2-clade mtDNA. Based on the bigger data set (n = 111), which includes all downloaded (and additional endemic) Turkish NE2 haplotypes (Figure 3) that were not found in our current samples and may also represent prehistorical/historical mtDNA samples [66,67], the respective percentage amounts to 72.97%. Given their frequency and wide geographical distribution, with occurrences also in E and SE Anatolia, the endemic haplotypes NE7 and haplotype NE9 are good candidates to have been present at the beginning of pig domestication in N Mesopotamia but, of course, the remaining 13 NE2 haplotypes (Figure 3) revealed presently or earlier [35] or the downloaded sequences (Supplementary Table S1) may have been involved in the early domestication process in the Middle East. In any case, we could not identify any of these lineages among the currently studied sequences of wild boars from Europe, which is consistent with the previous position that the Anatolian domestic pig lineages brought to Europe by Neolithic Anatolian settlers were largely replaced by European wild boar lineages [36]. At least so far, Middle Eastern NE2 lineages that were found in Neolithic pigs from Romania and wild boars from a pre-Neolithic cultural site in NE Italy [115,116] appear to be rare exceptions in Europe. Concordant with this, our present results do not favor the hypothesis of a late Pleistocene migration of NW Anatolian lineages to the Balkans. On the other hand, as to our knowledge no NE haplotypes were recorded from north of the Black Sea ([36], fig. 1A), which would represent a possible alternative late Pleistocene migration route from the Middle East to eastern and central Europe [117,118]. However, possible late Pleistocene Anatolian gene pool signals that may have existed for a short period of time after the Late Glacial Maximum in the Balkans might have been largely extinguished by massive increases and expansions of predominant European E1 haplotypes that would have existed in southeastern and eastern glacial refugia [50,117,118]. At least the haplotypes currently found in Turkish Thrace are widely distributed in eastern, southern, and southeastern European.

### 4.5. Phylogenetic and Spatial Patterns

The spatial distribution of haplotypes of some genetic clusters as revealed by our Geneland analysis (Figure 4) does not fully correspond to their phylogenetic positions: for instance, individual sequences of NE haplotype H4 were assigned to three different spatial clusters. According to our understanding this could be due to (1) the different sets of samples for the phylogenetic and the Geneland analyses, (2) the wide geographical distribution of some (introgressed) samples, which led to relatively low assignment probabilities and ambiguous cluster assignments in several cases, and (3) the generally shallow phylogenetic differentiation of all sequences. But as already found earlier [68], our phylogenetic results show three major phylogroups, the Asian, the Middle Eastern (NE1, NE2), and the European (E1, E2), and with the Asian phylogroup being tentatively subdivided into three haplogroups. However, no significant phylogenetic gaps occurred between any of them, which is due to the absence of longer lasting intraspecific breaks of mitochondrial genealogies due to long-term separate spatial distribution across the whole natural range of the species. In fact, the numbers of mutation steps between the three major phylogroups are negligible given the respective evolutionary divergence within each of the phylogroups as indicated in our network; indeed, only three mutation steps are present between the Asian and the NE phylogroup and the NE and the E phylogroup, respectively. Only three mutation steps are also present between the phylogroups NE1 and NE2, which suggests that the NE1 haplotypes from outside of Anatolia in the Middle East [68,70] exclusively comprise slightly more derived haplotypes than all NE2 haplotypes. Our network suggests a similar evolutionary scenario for the European E1 and E2 haplotypes and a similarly shallow evolutionary divergence pattern among the three Asian phylogroups (Figure 3). In fact, apart from potentially confusing introgressions resulting from anthropogenic translocations, all currently used samples in our ML and BI phylogenies (which force all haplotypes into bifurcating relationships) and in the network analysis (that allows for alternative evolutionary—reticulate—pathways) indicate continuous shallow mtDNA evolution across the whole range of the Palearctic distribution of wild boars.

Given the currently demonstrated absence of natural gene flow between NW Anatolia and the SE Balkans, the phylogenetic link (Figure 3) between SE Balkan E1 (H2, H93, H5) sequences with the NE2 sequences from SE and S Anatolia as well as Azerbaijan, Iran, Iraq, and Armenia (H13, H18, H148) is definitely unexpected, particularly because not a single wild boar from more western regions in Anatolia carried one of that latter NE2 haplotypes: this phylogeographic pattern, however, underpins our interpretation of no natural mitochondrial gene flow across the Pleistocene and early Holocene land bridge(s) that could have provided repeatedly migration, such as, for example, in brown hares, *Lepus europaeus*, [14,15,16,17,18,19,20,21,22,23] and other terrestrial mammal species ([10,11,12,13]). Rather, it corresponds to the phylogeographic or chorological pattern of other terrestrial species, like the badgers, *Meles meles* and *M. canescens* [10,11,12,13,24], that have their southeasternmost and westernmost distributions in Turkish Thrace and in NW Anatolia, respectively. However, even in brown hares only gene flow from NW Anatolia to the SE Balkans was observed [14,15,16,17,18,19,20,21,22,23] but not in the opposite direction. Future phylogeographic studies of additional terrestrial mammal species (and other terrestrial vertebrates) should widen our understanding of the evolutionary and biogeographical meaning of this region that has repeatedly connected Europe with SW Asia by Pleistocene land bridges. The mismatch between the geographical distribution of the currently observed mtDNA haplotypes in this region with their phylogenetic relationships may be explained by alternative patterns of spatial distribution and gene flow in wild boars during various phases of the late Pleistocene, including distribution and migration across regions east and north of the Black Sea, as suggested for instance for wild boars from north of the Caucasus and Italy [83] as well as possible repeated range expansions and contractions of Middle Eastern gene pools and intraspecific incomplete lineage sorting [119]. On the other hand, the similar (z = −1.561, *p* = 0.142, d.f. = 14, Mann–Whitney test) lineage diversity currently observed in whole Asia Minor (Shannon–Weaver Index = 1.37) compared to the small area of Turkish Thrace (Shannon–Weaver Index = 1.45), with eight and six haplotypes in the two regions, respectively, disregarding anthropogenic translocated E1 haplotypes in Anatolia, could be due to a higher dynamics of Balkan wild boar populations after the Late Glacial Maximum than within the Anatolian populations. This finding for wild boars of the SE Balkans/NW Anatolian biogeographic crossroads is, for instance, in contradiction to that of brown hares, which show higher diversity in Asia Minor than in Europe [14,15,16,17,18,21]. However, for both the Anatolian data set, the larger data set of Middle Eastern lineages, as well as for the species-wide mtDNA lineage system, the general phylogeographic pattern is consistent with a combination of phylogeographic categories III and IV of Avise [119], namely shallow gene trees with sympatric and allopatric lineage distribution in Anatolia and with more allopatric lineages for the Middle East and even more allopatric lineages for the whole species range.

## 5. Conclusions

Our currently studied wild boar samples and our previously published [35] samples from Anatolia revealed D-loop haplotypes both endemic to the Anatolian Peninsula and more widely distributed in the Middle East, as well as a few E1 haplotypes, but no E2 haplotypes and no haplotypes typical for “Asia”, i.e., from Central Asia, China, the Far East, or Southeast Asia, with very similar phylogenetic partitioning into clades (phylogroups) as already reported earlier [35,68]. Our phylogeographic analyses of the current and previously published Turkish haplotypes and those from other parts of the Middle East as well as from Europe and the Far East indicated ancient and/or historical translocations of pigs and/or wild boars of European origin to Anatolia (and other regions of the Middle East) in line with previous studies. Consistent with previous hypotheses, no NE1 haplotypes were found in Anatolia, and no ancient or modern mitochondrial gene flow from northwestern Anatolia to Europe via the Bosphorus/Sea of Marmara/Dardanelles, which has represented a migration barrier for many terrestrial mammals since the breakup of the land bridge in the mid-Holocene, has been detected. However, the absence of a signal of mitochondrial gene flow during the late Pleistocene and early Holocene remains remarkable, given that some NE haplotypes were found earlier in pre-Neolithic and Neolithic samples of Romania and NW Italy [115,116]. The spatial distribution of E1 lineages in S and NW Anatolia may suggest historical anthropogenic translocations of pigs from Europe (especially Italy) to the medieval Crusader states and the Empire of Trebizond, respectively. All these medieval states were colonized by European settlers (mostly Italians, French, Franks/Germans, and English) and were supplied through a dense European trade network, particularly of Italian (Venetian, Genoese, and Pisan) merchants.

Our phylogenetic analyses of all our currently produced and previous sequences downloaded from GenBank revealed close phylogenetic connections between the NE1 and NE2 clades, the Asian and NE clades, and the NE and E clades, respectively. They suggest that the NE1 clade was somewhat more modern than the NE2 clade, contrary to an earlier network suggestion [68]. The phylogenetic positions of the NW Anatolian haplotypes and the SE Balkan haplotypes, specifically those from Turkish Thrace, do not indicate phylogenetic continuation, highlighting the absence of ancient or modern gene flow from NW Anatolia to SE Europe. This spatial and phylogenetic mismatch of haplotypes does not support the hypothesis that the Anatolian Peninsula could have served as a source of (mitochondrial) genetic diversity for potential refugial wild boar populations in the SE and S Balkans during the Late Glacial Maximum. More complex population dynamics in the Anatolian Peninsula and the Middle East, in general, during the late Pleistocene, with unrecognized advances and retractions of regional wild boar populations due to variable climatic conditions, may have led to the currently observed phylogenetic discordance of haplotypes in NW Anatolia and the SE Balkans. A more comprehensive set of geographical samples, particularly from eastern Anatolia and from east of the Black Sea, as well as nuclear gene pool data may help to elucidate the population dynamics and distribution patterns of wild boars in the late Pleistocene in the Anatolian Peninsula and other regions of the Middle East, and possibly also to understand the migratory relationships between Middle Eastern and eastern European populations during the late Pleistocene. The different external phenotypes observed in Anatolian wild boars may reflect variable portions of historically introgressed (European) pig gene pools, which could be further studied by nuclear gene pools and specifically also by coat color gene (e.g., MC1R, ASIP) variation.

## Figures and Tables

**Table 1:** Genetic diversity indices and neutrality test results based on mitochondrial D-Loop sequences for all data, i.e., those that have resulted in seven clades (NE1—Near East1, NE2—Near East2, E1—Europe2, E2—Europe-1, Clade 1, Clade 2, Asian Clade 3) in our phylogenetic analyses.

Group	N	S	PIS	H	Hd	π	k	θ_w_	Tajima’s D	Fu’s F
NE1	26	7	5	6	0.612	0.0049	1.815	0.0049	−0.0317	0.3713
NE2	173	14	11	16	0.728	0.0066	2.450	0.0066	0.0057	−1.1751
E1	1238	55	34	88	0.886	0.0059	2.100	0.0201	−1.8657 **	−26.0087 **
E2	38	6	2	7	0.659	0.0023	0.866	0.0038	−1.0617	−2.8855 *
Clade 1	20	23	19	15	0.958	0.0232	8.553	0.0176	1.2713	−3.1207
Clade 2	4	8	1	3	0.833	0.0112	4.167	0.0117	−0.44637	1.3991
Asian Clade 3	347	52	30	72	0.928	0.0085	3.092	0.0223	−1.6874 **	−25.4988 **
Total	1846	93	66	202	0.943	0.0170	5.954	0.0328	−1.2897 *	−23.8224 **

Statistical significance: * *p* < 0.05, ** *p* < 0.01. N: number of sequences examined; S: number of polymorphic (segregating) sites; PIS: number of parsimony informative sites; H: number of haplotypes; Hd: haplotype diversity; π: nucleotide diversity; k: average number of nucleotide differences; θ_w_: Watterson’s estimator theta (per site).

**Table 2:** Analysis of molecular variance (AMOVA) for the seven phylogenetic clusters.

Source of Variation	**df**	**Sum of** **Squares**	**Variance** **Components**	**Percentage** **of Variation**	**Significance** **(*p*)**
between clades	6	3943.824	4.21882	76.13	<0.0001
within clades	1839	2432.621	1.32280	23.87	<0.0001
total	1845	6376.445	5.54162		
Fixation index *F_ST_* = 0.76130					

df, degree of freedom.

## Data Availability

The currently obtained new mtDNA sequences have been deposited on GenBank under the accession numbers provided in Supplementary Table S1.

## Supplementary Materials

The following supporting information can be downloaded at https://www.mdpi.com/article/10.3390/ani15131828/s1, the list of samples and associated details are in Supplementary Table S1: List of Türkiye samples presently sequenced and studied earlier [35], as well as downloaded sequences for the present combined phylogenetic analyses. The ML phylogenetic tree is in supplementary Figure S1. References [35,50,65,66,67,68,70,79,103,120,121,122,123,124,125,126,127] in Supplementary Materials.

## Abbreviations

The following abbreviations are used in this manuscript:

B.P.	Before present
YBP	Years before present
A.D.	Anno Domini
PCR	Polymerase chain reaction
D-loop	Displacement loop (of mtDNA)
mtDNA	Mitochondrial deoxyribonucleic acid
