# Supplementary material for: Mitochondrial Phylogeography of Wild Boars, Sus scrofa, from Asia Minor: Endemic Lineages, Natural Immigration, Historical Anthropogenic Translocations, and Possible Introgression of Domestic Pigs
Source: Animals (Basel). 2025 Jun 20;15(13):1828. doi: 10.3390/ani15131828 (PMC12248688; doi:10.3390/ani15131828)
Supplement: Supplementary file 1 [file animals-15-01828-s001.zip › Supplementary Figure S1.pdf]

Supplementary Figure S1.

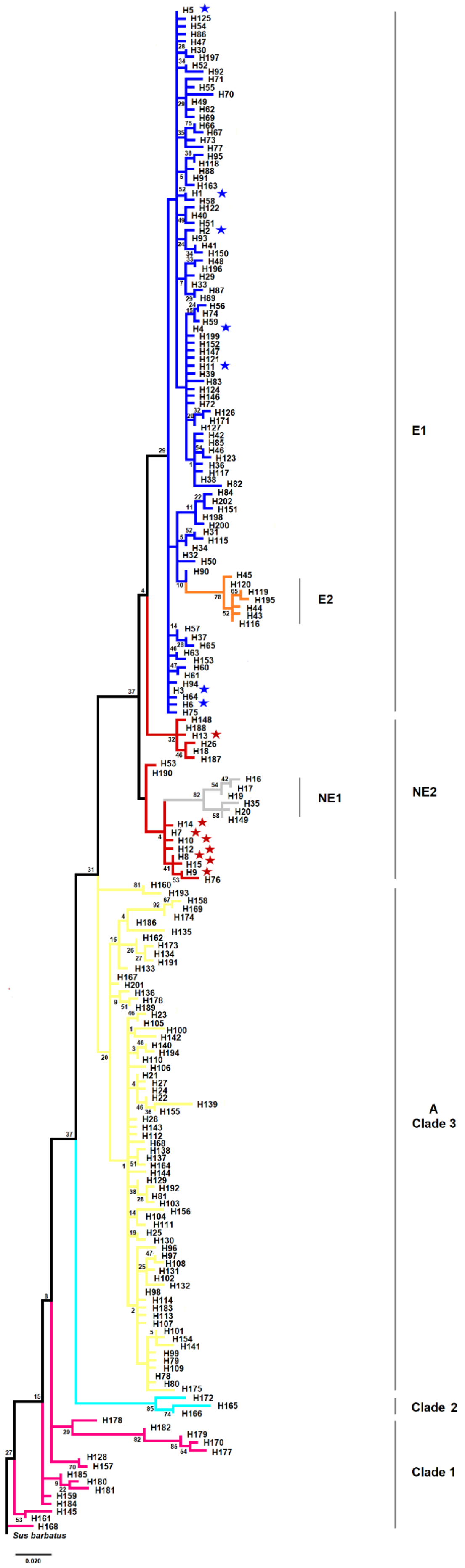

ML phylogenetic tree. Haplotype codes correspond to the ones mentioned in the text and given in supplementary Table S1. Asterisks denote haplotypes from Türkiye.
